# Effects of Post Annealing on Electrical Performance of Polycrystalline Ga_2_O_3_ Photodetector on Sapphire

**DOI:** 10.1186/s11671-020-03324-x

**Published:** 2020-05-07

**Authors:** Haodong Hu, Yuchen Liu, Genquan Han, Cizhe Fang, Yanfang Zhang, Huan Liu, Yibo Wang, Yan Liu, Jiandong Ye, Yue Hao

**Affiliations:** 1 State Key Discipline Laboratory of Wide Band Gap Semiconductor Technology, School of Microelectronics, Xidian University, Xi’an, 710071 China; 2 School of Electronic Science and Engineering, Nanjing University, Nanjing, 210093 China

**Keywords:** Gallium oxide (Ga_2_O_3_), Post annealing, Solar-blind, Ultraviolet, Photodetector

## Abstract

Effects of post annealing on the physical and electrical properties of solar-blind polycrystalline gallium oxide (Ga_2_O_3_) ultraviolet photodetectors on the sapphire substrate are investigated. The grain size of poly-Ga_2_O_3_ becomes larger with the post annealing temperature (PAT) increasing from 800 °C to 1000 °C, but it gets smaller with further raising PAT to 1100 °C. A blue shift is observed at the absorption edge of the transmittance spectra of Ga_2_O_3_ on sapphire as increasing PAT, due to the incorporation of Al from the sapphire substrate into Ga_2_O_3_ to form (Al_*x*_Ga_1–*x*_)_2_O_3_. The high-resolution X-ray diffraction and transmittance spectra measurement indicate that the substitutional Al composition and bandgap of (Al_*x*_Ga_1–*x*_)_2_O_3_ annealed at 1100 °C can be above 0.30 and 5.10 eV, respectively. The *R*_max_ of the sample annealed at 1000 °C increases about 500% compared to the as-deposited device, and the sample annealed at 1000 °C has short rise time and decay time of 0.148 s and 0.067 s, respectively. This work may pave a way for the fabrication of poly-Ga_2_O_3_ ultraviolet photodetector and find a method to improve responsivity and speed of response.

## Background

Deep ultraviolet (DUV) solar-blind photodetectors have a wide range of applications such as monitoring ozone holes and detecting flames with the inherent advantage of strong anti-interference ability [1]. Compared with traditional semiconductor materials like silicon and germanium, wide bandgap semiconductor materials are considered to be ideal materials for solar-blind photodetectors which have better selectivity for ultraviolet light and better adaptability in harsh environments [2]. Lots of researchers have been focused on AlGaN, MgZnO, and Ga_2_O_3_ DUV solar-blind photodetectors [2–4]. Ga_2_O_3_ attracts great attention due to its superior optical properties, chemical stability, and high strength with a bandgap of 4.8 eV, which is a promising material for solar-blind photodetectors [5–13]. Ga_2_O_3_ thin films have been obtained on foreign substrates by molecular beam epitaxy (MBE) [5, 6], radio-frequency magnetron sputtering (RFMS) [7], pulsed laser deposition (PLD) [8, 9], atomic layer deposition (ALD) [10], halide vapor phase epitaxy (HVPE) [11], metal-organic chemical vapor deposition (MOCVD) [12], and sol-gel method [13]. Among these methods, RFMS deposition has been widely used to fabricate various films due to its advantages of easy controllability, high efficiency, harmless, and low cost. Therefore, we used this method to grow Ga_2_O_3_ thin films for DUV solar-blind photodetectors.

In this work, poly-Ga_2_O_3_ solar-blind photodetectors were fabricated on the sapphire substrate. It is demonstrated that the Al atoms are incorporated from the sapphire substrate into Ga_2_O_3_ to form (Al_*x*_Ga_1–*x*_)_2_O_3_ after post thermal annealing. The structural properties, substitutional Al composition *x*, optical properties, and photodetector performance of poly-(Al_*x*_Ga_1–*x*_)_2_O_3_ films with different post annealing temperatures (PATs) were investigated.

## Method

**Fig. 1:**
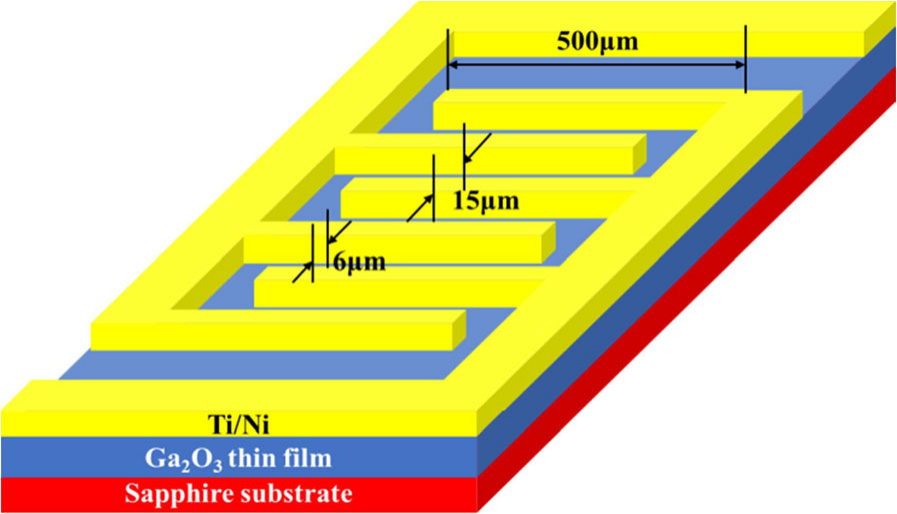
The schematic of the photodetector based on poly-Ga_2_O_3_ thin film

In this experiment, poly-Ga_2_O_3_ thin films were grown on single-polished (0006)-oriented sapphire substrates by RFMS at 600 °C with the sputtering power of 120 W. The working pressure was kept constant at 5 mTorr and the flow of argon was 20 sccm throughout the deposition. The thickness of the films deposited on sapphire was measured to be around 164 nm. After the deposition, post thermal annealing was carried out in an air atmosphere for 1 h at 800 °C, 900 °C, 1000 °C, and 1100 °C. After annealing, the samples were cooled to room temperature with a speed of 100 °C/min. The 30 nm Ti and 80 nm Ni were then deposited by magnetron sputtering as an electrode. After the interdigital electrode patterning and etching, the metallic contacts on Ga_2_O_3_ were formed by the rapid thermal annealing at 470 °C in a nitrogen atmosphere [14]. The fabricated poly-Ga_2_O_3_ solar-blind photodetectors have metal-semiconductor-metal (MSM) interdigital electrodes as shown in Fig. 1. The length, width, and space between the fingers were 500 μm, 6 μm, and 15 μm, respectively, and the total length of the fingers is 1.8 cm.

## Results and Discussion

**Fig. 2:**
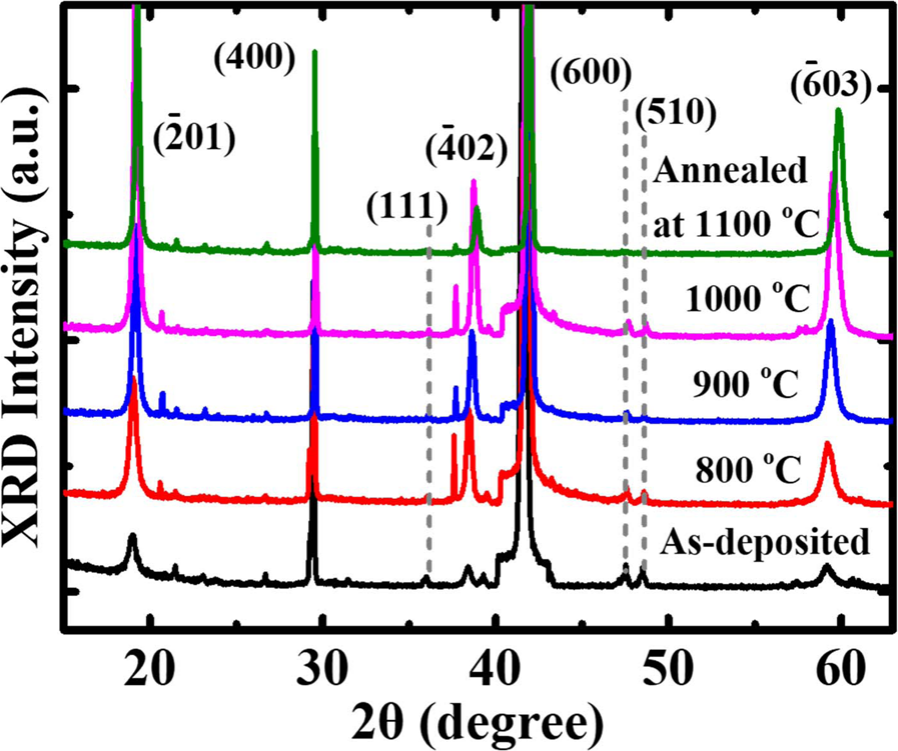
The XRD peaks of the samples without and with post thermal annealing at different temperatures

The structural properties of the Ga_2_O_3_ films were investigated by high-resolution X-ray diffraction (HRXRD). Figure 2 presents the HRXRD curves for the samples that as-deposited and annealed at different temperatures. Peaks corresponding to $\left(\overline{2}01\right)$, (400), (111), $\left(\overline{4}02\right)$, (600), (510), and $\left(\overline{6}03\right)$ planes of β-Ga_2_O_3_ crystals [15] reveal that the Ga_2_O_3_ film consists of monoclinic β-Ga_2_O_3_ polycrystalline with random orientation. The as-deposited sample exhibits a higher peak intensity for the (400) plane compared to the other planes. The PAT leads to the improvement of the intensities of $\left(\overline{2}01\right)$, (400), $\left(\overline{4}02\right)$, and $\left(\overline{6}03\right)$ planes.

**Fig. 3:**
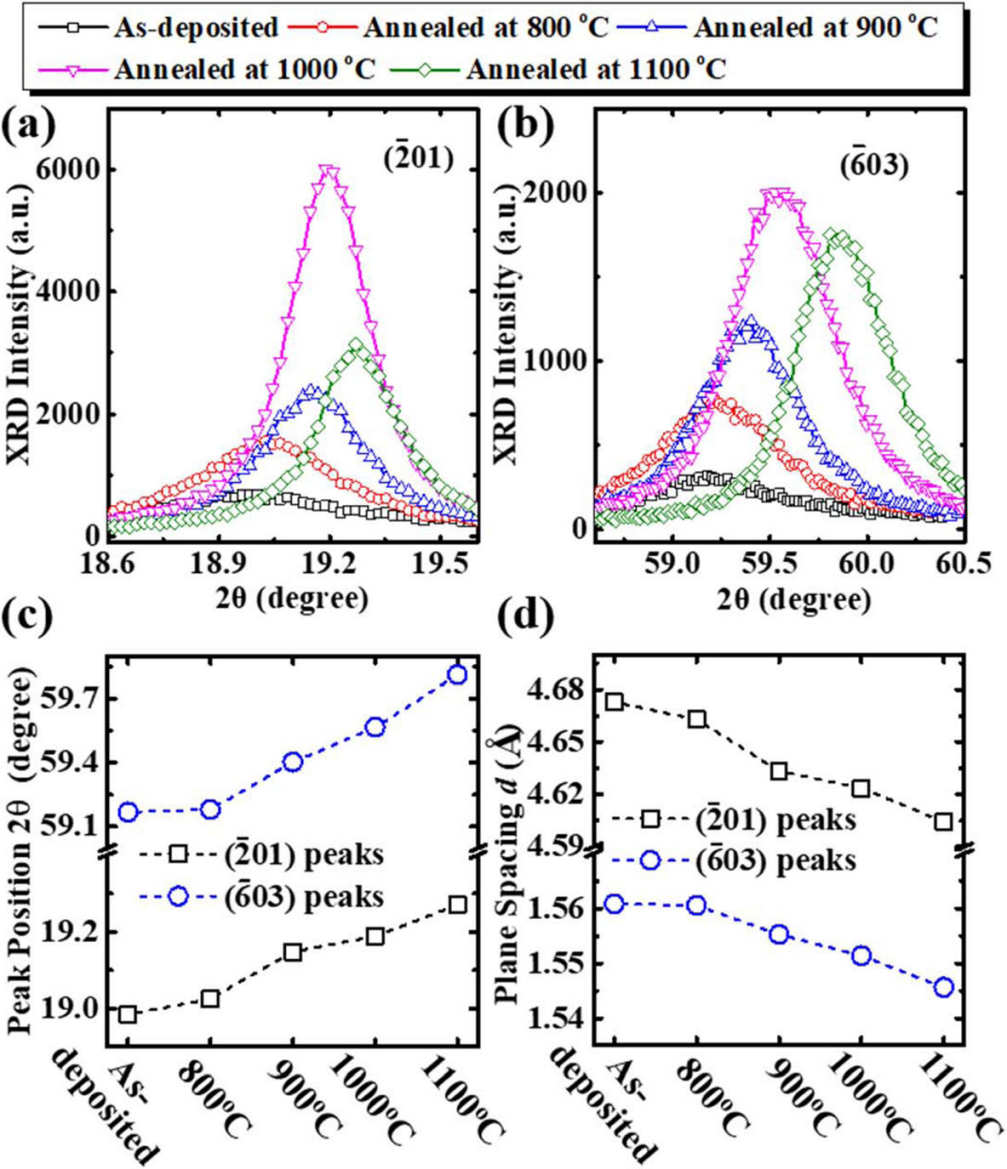
The XRD peaks of **a**$\left(\overline{2}01\right)$ plane and **b**$\left(\overline{6}03\right)$ plane of the samples before and after annealing. **c** peak position and **d** plane spacing of $\left(\overline{2}01\right)$ and $\left(\overline{6}03\right)$ planes

Figure 3a and b focus on the HRXRD peaks for $\left(\overline{2}01\right)$ and $\left(\overline{6}03\right)$ planes, respectively. The full width at half maximum (FWHM) of the peak was used to calculate the grain size by solving the Debye-Scherrer formula [16] to evaluate the dependence of the crystalline quality of Ga_2_O_3_ films on PAT. It can be seen from Table 1 that higher annealing temperature yields larger grain size as PAT increases from 800 °C to 1000 °C, but the grain size decreases slightly at the PAT of 1100 °C. The diffusion of Al from the Al_2_O_3_ substrates into Ga_2_O_3_ films underwent a PAT above 1000 °C has been widely observed [17–19]. As shown in Fig. 3c, the peaks of HRXRD shifting to the higher diffraction angle is due to that Al from the sapphire substrate diffuses into Ga_2_O_3_ film to form (Al_*x*_Ga_1–*x*_)_2_O_3_ after annealing.

**Table 1:** The grain size of polycrystalline films at different annealing temperatures

Temperature (°C)	FWHM (°)	Grain size (nm)
**As-deposited**	0.49135	15.99
**800**	0.47789	16.22
**900**	0.37031	20.93
**1000**	0.28513	27.18
**1100**	0.29602	26.18

Based on the Bragg’s law, the plane spacing *d* of $\left(\overline{2}01\right)$ and $\left(\overline{6}03\right)$ planes of (Al_*x*_Ga_1–*x*_)_2_O_3_ are calculated and shown in Fig. 3d, respectively. According to Ref. [20], the lattice parameters can be calculated by *a* = (12.21 − 0.42*x*) Å, *b* = (3.04 − 0.13*x*) Å, *c* = (5.81 − 0.17*x*) Å, *β* = (103.87 + 0.31*x*)°. The *d* of $\left(\overline{6}03\right)$ is expressed as [21]

1 $$ \frac{1}{d^2}=\frac{h^2}{a^2{\sin}^2\beta }+\frac{k^2}{b^2}+\frac{l^2}{c^2{\sin}^2\beta }-\frac{2 hl\cos \beta }{ac\sin^2\beta }, $$

where *h* = -6, *k* = 0, and *l* = 3. Based on the values in Fig. 3d, the *x* of poly-(Al_*x*_Ga_1–*x*_)_2_O_3_ can be achieved. The bandgap *E*_g_ of (Al_*x*_Ga_1–*x*_)_2_O_3_ can be calculated by

2 $$ {E}_{\mathrm{g}}(x)=\left(1-x\right){E}_{\mathrm{g}}\left[{Ga}_2{O}_3\right]+{xE}_{\mathrm{g}}\left[{Al}_2{O}_3\right]- nx\left(1-x\right), $$

where *E*_g_ [Ga_2_O_3_] = 4.65 eV, *E*_g_ [Al_2_O_3_] = 7.24 eV, n = 1.87 eV [22]. The calculated *x* and *E*_g_ values of the poly-(Al_*x*_Ga_1–*x*_)_2_O_3_ are shown in Table 2. An *x* value above 0.30 is achieved in the sample after a PAT at 1100 °C.

**Table 2:** Comparison of the calculated Al content and *E*_g_ of poly-(Al_*x*_Ga_1–*x*_)_2_O_3_ after thermal annealing according to HRXRD in Fig. 3 and experimental results of transmittance spectra

	800 °C	900 °C	1000 °C	1100 °C
**Substitutional Al composition**	0.02	0.14	0.22	0.35
**Calculated*E***_**g**_	4.67 eV	4.79 eV	4.90 eV	5.13 eV
**Experimental*E***_**g**_	4.72 eV	4.78 eV	4.81 eV	5.10 eV

**Fig. 4:**
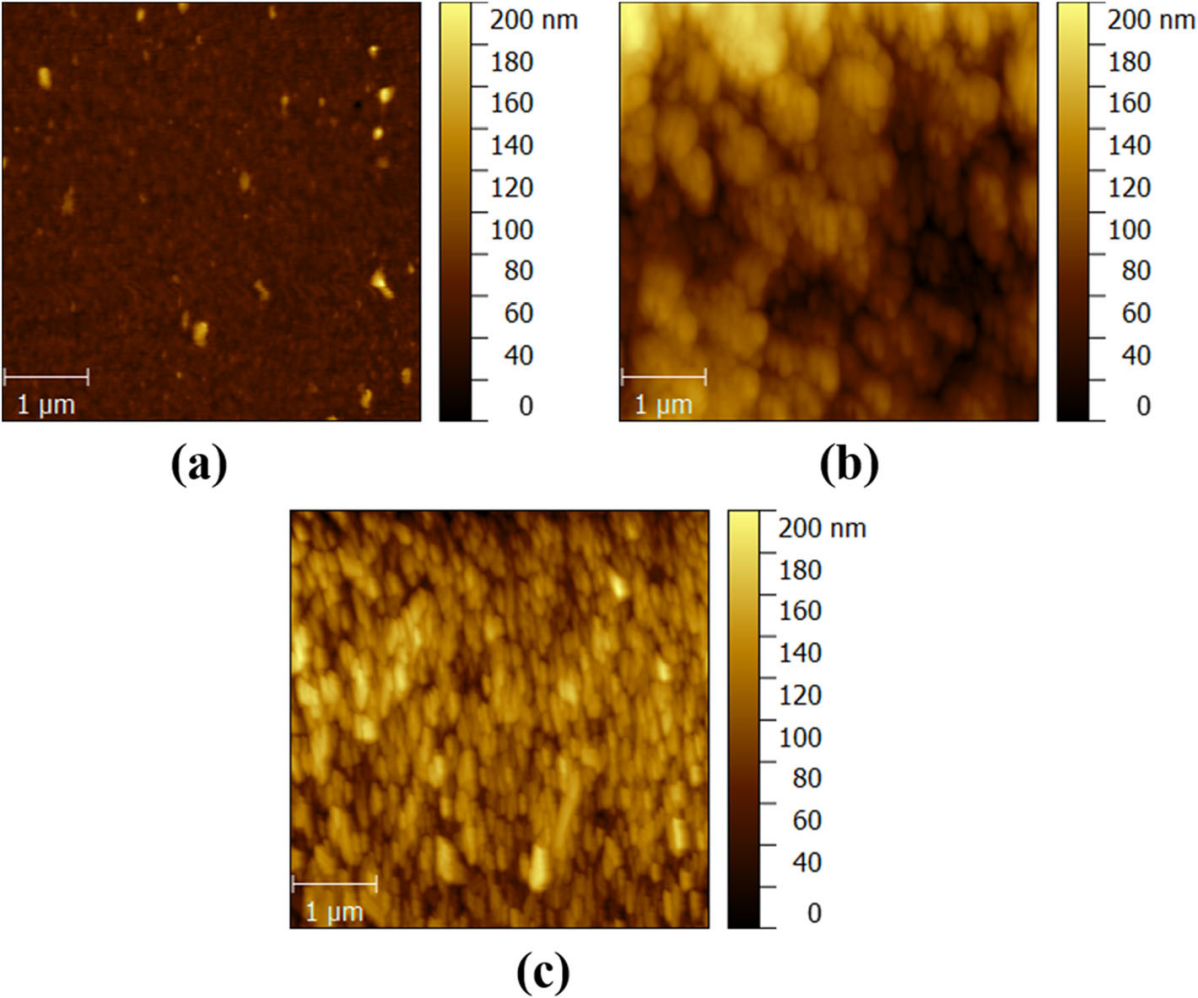
AFM images of **a** as-deposited poly-Ga_2_O_3_ on sapphire, **b** samples annealed at 800 °C, and **c** 900 °C

Atomic force microscope (AFM) images in Fig. 4 show that the surface root-mean-square (RMS) roughness values of the as-deposited film and the samples annealed at 800 °C and 900 °C are 3.62 nm, 10.1 nm, and 14.1 nm, respectively. The recrystallization caused by the high PAT results in a larger grain size, which can be additionally confirmed by a rougher surface.

**Fig. 5:**
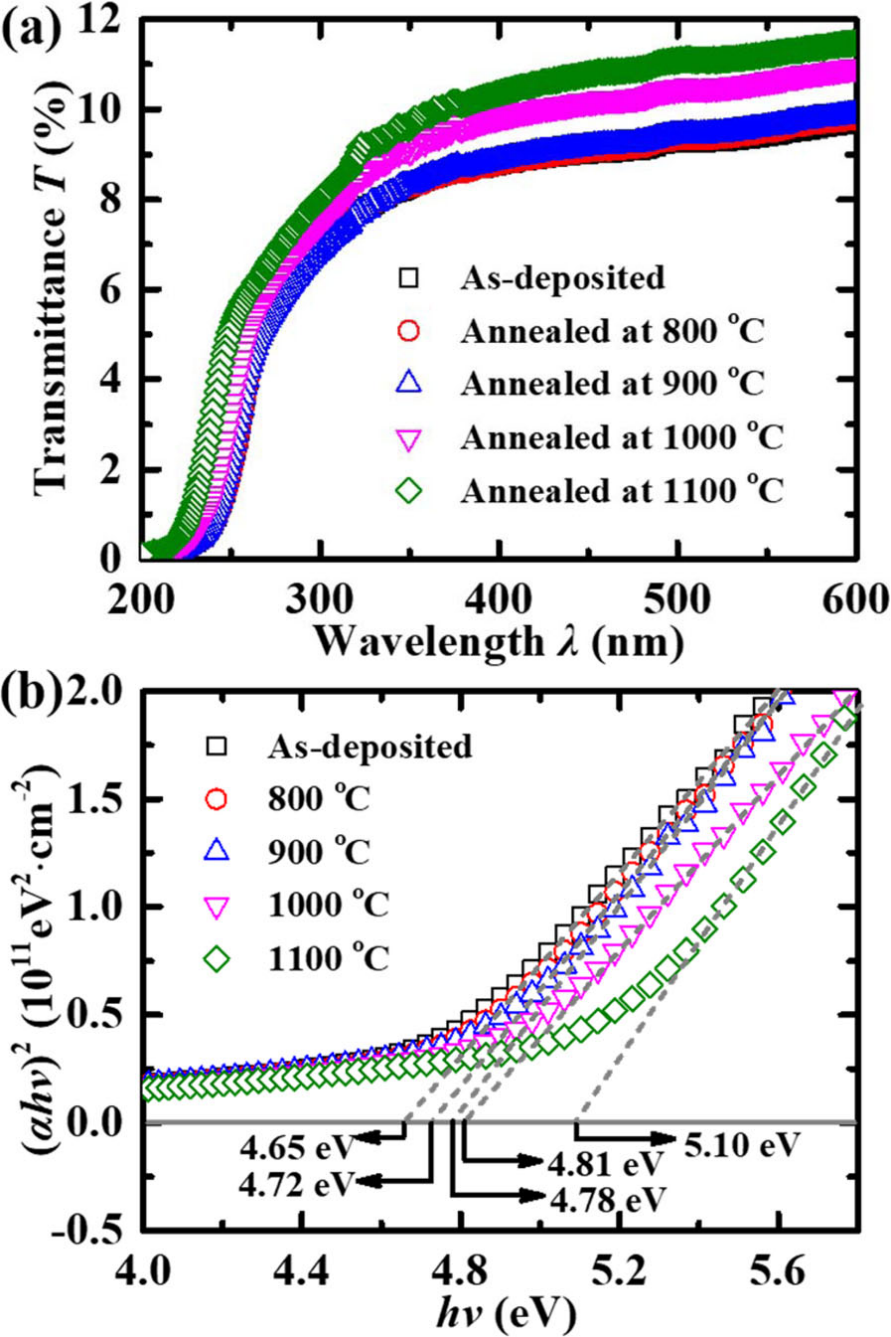
**a** Transmittance spectra of as-deposited and annealed poly-(Al_*x*_Ga_1–*x*_)_2_O_3_ samples **b** (*α*h*ν*)^2^ vs. h*ν* curves for poly-Ga_2_O_3_ samples. The extrapolation of the linear regions to the horizontal axis estimates the *E*_g_ values

The values of *E*_g_ of the (Al_*x*_Ga_1–*x*_)_2_O_3_ thin films before and after annealing were characterized by measuring the transmittance spectra. As shown in Fig. 5a, the annealed samples have a blue shift at the absorption edge compared to the as-deposited one. A shorter *λ* is acquired with the increase of PAT, due to the incorporation of Al. The Ga_2_O_3_ samples have a very low transmittance even in the visible range, which might be due to the nonradiative complex absorption induced by the defects in the materials. The absorption coefficient *α* of the films is calculated by [23, 24]

3 $$ \alpha =\left(1/t\right)\ln \left[{\left(1-r\right)}^2/T\right], $$

where *T* is the transmittance, *r* is the reflectance, and *t* is the film thickness. The relation between absorption coefficient *α* and incident photon energy h*ν* follows a power law of the form

4 $$ \left(\alpha h\nu \right)=B{\left( h\nu -{E}_{\mathrm{g}}\right)}^{1/2}, $$

where *B* is the absorption edge width parameter [23]. By using these formulas, the relationship between h*ν* and (*α*h*ν*)^2^ can be obtained as shown in Fig. 5b. By extrapolating the linear regions of the plot to the horizontal axis, the *E*_g_ values of the samples are evaluated as 4.65 eV, 4.72 eV, 4.78 eV, 4.81 eV, and 5.10 eV. As shown in Table 2, the experimental *E*_g_ values of the samples are consistent with those calculated based on the HRXRD results.

**Fig. 6:**
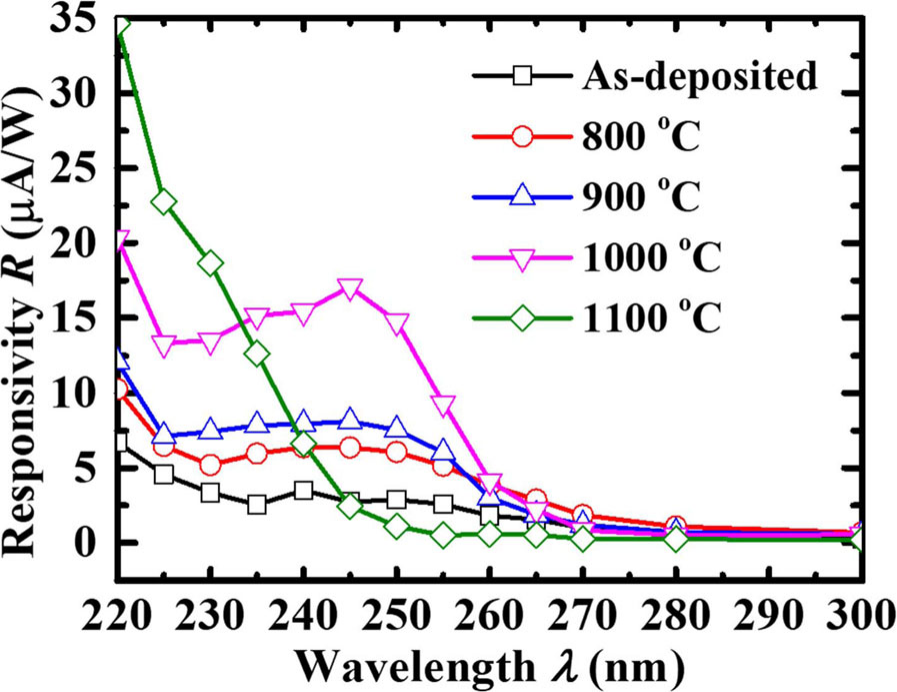
*R* versus illumination optical *λ* for the poly-(Al_*x*_Ga_1–*x*_)_2_O_3_ photodetectors at *V*_bias_ of 5 V

To investigate the responsivity *R* and photocurrent *I*_photo_ of poly-(Al_*x*_Ga_1–*x*_)_2_O_3_ photodetectors, optical measurements varied different illumination *λ* from 220 to 300 nm with a *P*_light_ of 0.5 mW/cm^2^. The *R* is calculated by

5 $$ R=\left({I}_{\mathrm{photo}}-{I}_{\mathrm{dark}}\right)/\left({P}_{\mathrm{light}}S\right), $$

where *I*_dark_ is the dark current and *S* is the effective illuminated area. Figure 6 shows a visible blue shift in maximum *R* of the annealed samples compared to the as-deposited film. This proves that a larger *E*_g_ of polycrystalline samples has been obtained after annealing with the diffusion of Al from the sapphire substrate into Ga_2_O_3_ to form (Al_*x*_Ga_1–*x*_)_2_O_3_. The *R*_max_ of the device annealed at 1100 °C is 35 μA/W, which is smaller than the 0.037 A/W, 0.903 A/W, and 1.13 mA/W those were grown by MBE [5], PLD [25], and sol-gel method [26], respectively, due to the fact that the poly-Ga_2_O_3_ has a low transmittance, as shown in Fig. 5a. But compared to the as-deposited device, the *R*_max_ of the device annealed at 1000 °C increases by about 500%. It is noted that *R* of devices decreases at wavelength shorter than that at *R*_max_, similar to that in [27]. This could be due to the energy loss occurs during the relaxation process of carriers in case of photon energy above *E*_g_ of materials. *R*_max_ increasing with the PAT rising from 800 °C to 1000 °C is attributed to the increased grain size of the film.

**Fig. 7:**
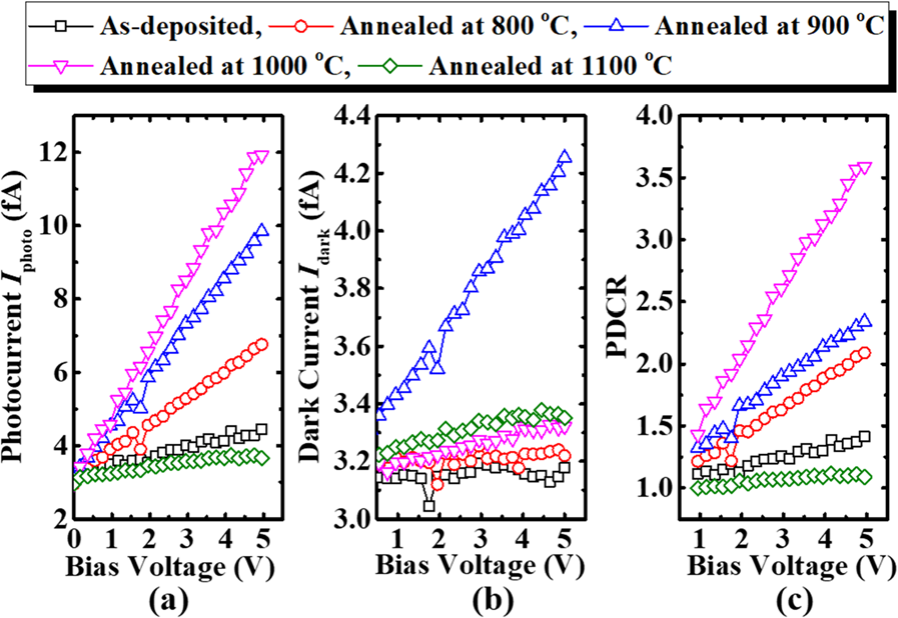
**a***I*_photo_-*V*_bias_, **b***I*_dark_-*V*_bias_, and **c** PDCR characteristics of the as-deposited poly-(Al_*x*_Ga_1–*x*_)_2_O_3_ film and the samples annealed at different temperatures under the illumination intensity of 0.5 mW/cm^2^ and *λ* of 254 nm

Figure 7 shows the photocurrent *I*_photo_, dark current *I*_dark_, and PDCR versus bias voltage *V*_bias_ for the photodetectors under the illumination intensity of 0.5 mW/cm^2^ and *λ* of 254 nm. As shown in Fig. 7a, *I*_photo_ increases almost linearly with the *V*_bias_. Furthermore, as PAT raises from 800 °C to 1000 °C, photodetectors gain a larger *I*_photo_. But the *I*_photo_ of the device annealed at 1100 °C is lower than that of the as-deposited sample, due to the energy of the photon is less than bangap of the sample annealed at 1100 °C, which cannot generate photo-carriers. The annealed samples show a higher *I*_dark_ than the as-deposited sample, as depicted in Fig. 7b. It is speculated that the recrystallization enhances the conductivity of poly-Ga_2_O_3_, resulting in the enhancement of both *I*_photo_ and *I*_dark_ of the photodetectors, and the PDCR of the sample with a PAT of 1000 °C is higher than those of the other samples. It can be noted that the dark current of the sample annealed at 900 °C is larger than others, which may be ascribed to the increased carriers with the PAT increasing, but with the PAT further increasing, interdiffusion of the Al and Ga takes place on a sapphire substrate, thus destroying the conductivity of the film [17].

**Fig. 8:**
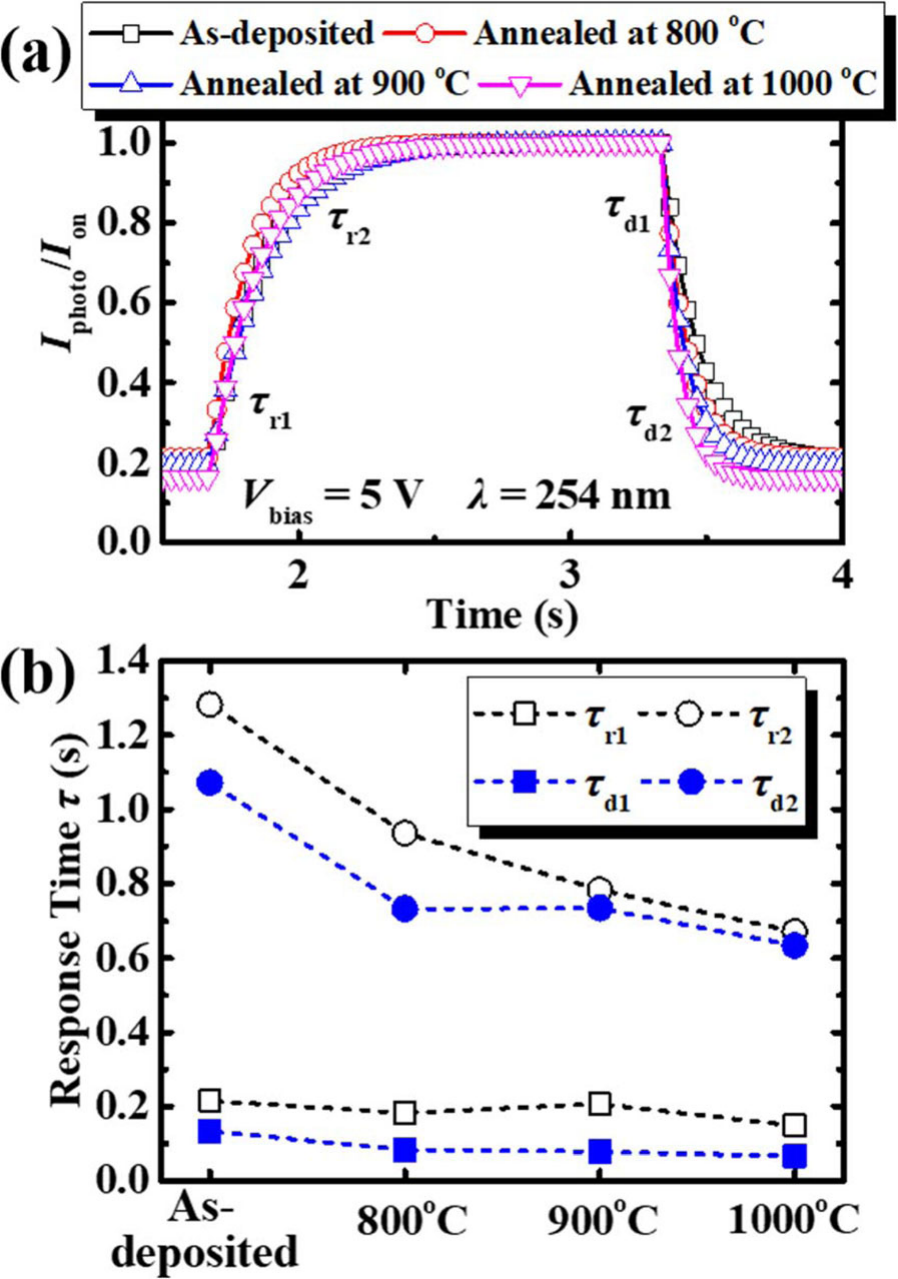
**a** Time-dependence of photoresponse characteristics **b** rise and decay time

The photoresponse characteristics of the photodetectors are depicted in Fig. 8a. An illumination with *λ* of 254 nm was used during the measurements. The *P*_light_, *V*_bias_, and period were 0.5 mW/cm^2^, 5 V, and 5 s, respectively. There are two procedures of rising and decaying processes: fast-response and slow-response. Generally, the fast-response component can be attributed to the rapid change of carrier concentration as soon as the light is turned on/off [28], while the photo-generated carriers might be trapped by the defect levels in the bandgap, which could delay the carrier collection during the UV illumination and recombination as the light was turned off, resulting in the slow-response component. For a quantitative comparison study of the photodetector annealed at the different temperatures, the rise and decay processes can be fitted with a biexponential relaxation equation of the following type [29]:

6 $$ I={I}_0+{Ce}^{-t/{\tau}_1}+{De}^{-t/{\tau}_2}, $$

where *I*_0_ is the steady-state photocurrent, *t* is the time, *C* and *D* are the constant, *τ*_1_ and *τ*_2_ are two relaxation time constants. The rise time *τ*_r1_ and *τ*_r2_ correspond to the fast-response and the slow-response, respectively, and the decay time *τ*_d1_ and *τ*_d2_ of each photodetector are calculated, as shown in Table 3. It is clearly seen that the response time decreases after the annealing process. The rise time *τ*_r1_ is reduced from 0.215 s to 0.148 s, and the decay time *τ*_d1_ is reduced from 0.133 to 0.067 s. It is ascribed to the fact that the annealing process reduces the oxygen vacancies concentration in the poly-Ga_2_O_3_ film [28]. The direct transition becomes the main source of photo-generated unbalanced carriers, thereby the fast-response time decreases. The decay time *τ*_d2_ decreases from 1.072 to 0.634 s, indicating that there are fewer oxygen vacancies and other defects in the annealed samples as well, due to the time constant of the transient decay is generally governed by these traps. Further, the increased grain size with PAT can reduce the photo-carriers transportation time, improving the relaxation time properties of the devices.

**Table 3:** The rise time and decay time of UV photodetectors without post annealing and after the annealing at different temperatures

Temperature (°C)	***τ***_**r1**_ (s)	***τ***_**r2**_ (s)	***τ***_**d1**_ (s)	***τ***_**d2**_ (s)
**As-deposited**	0.215	1.283	0.133	1.072
**800**	0.183	0.936	0.083	0.733
**900**	0.207	0.783	0.078	0.735
**1000**	0.148	0.672	0.067	0.634

Table 4 shows the comparison of the *I*_dark_, rise time (*τ*_r_), and decay time (*τ*_d_) of solar-blind photodetectors based on β-, α-, and ε-Ga_2_O_3_ thin films synthesized by RFMS [30] and other techniques [2, 6, 26, 31–34]. As seen, the device has both low dark current and fast response time is difficult, but the photodetector we fabricated presents the low dark current and fast response time.

**Table 4:** The comparison of the *I*_dark_, rise time (*τ*_r_) and decay time (*τ*_d_) of solar-blind photodetectors based on β-, α-, and ε-Ga_2_O_3_ thin films synthesized by different techniques

Material	Method	***I***_**dark**_ (nA)	Rise time ***τ***_**r**_ (s)	Decay time ***τ***_**d**_ (s)	Ref.
Poly-Ga_2_O_3_	RFMS	0.0033 (5 V)	0.148/0.672	0.067/0.634	This work
β-Ga_2_O_3_	RFMS	0.11 (10 V)	0.31/1.52	0.05/0.91	[30]
β-Ga_2_O_3_	Laser MBE	80 (10 V)	0.86	1.02/16.61	[6]
β-Ga_2_O_3_	MBE	4 (20 V)	3.33	0.4	[31]
β-Ga_2_O_3_	PLD	430 (20 V)	0.87/10.81	0.54/13.98	[2]
β-Ga_2_O_3_	MOCVD	34 (10 V)	0.48	0.18	[32]
β-Ga_2_O_3_	Sol-gel	0.758 (30 V)	0.1/0.18	0.1/1.85	[26]
α-Ga_2_O_3_	MOCVD	8.1×10^−5^ (12 V)	Not given	0.042	[33]
ε-Ga_2_O_3_	MOCVD	0.037 (200 V)	2.5	0.4/2.6	[34]

## Conclusions

In summary, we deposited poly-Ga_2_O_3_ thin film by magnetron sputtering on the c-plane sapphire substrate with post thermal annealing under different temperature; then, the ultraviolet poly-Ga_2_O_3_ photodetector was fabricated. Compared to the as-deposited Ga_2_O_3_ thin film, the annealed samples possess a larger grain size and a wider bandgap due to the recrystallization and the diffusion of the Al into Ga_2_O_3_. The *R*_max_ of the device annealed at 1000 °C increases about 500% compared to the as-deposited device, and the sample annealed at 1000 °C shows a low dark current of 0.0033 nA under the bias of 5 V. Furthermore, the solar-blind photodetector fabricated on the film annealed at 1000 °C shows fast response time, with a rise and decay time of 0.148 s and 0.067 s, respectively. These results are useful to fabricate the DUV photodetectors with low dark current and fast response time.

## Data Availability

The datasets supporting the conclusions of this article are included within the article.

## Abbreviations

Ga_2_O_3_: Gallium oxide
PAT: Post annealing temperature
DUV: Deep ultraviolet
MBE: Molecular beam epitaxy
RFMS: Radio-frequency magnetron sputtering
PLD: Pulsed laser deposition
ALD: Atomic layer deposition
HVPE: Halide vapor phase epitaxy
MOCVD: Metal-organic chemical vapor deposition
MSM: Metal-semiconductor-metal
HRXRD: High-resolution X-ray diffraction
FWHM: Full width at half maximum
AFM: Atomic Force Microscope
RMS: Root-mean-square
